# Acute Pancreatitis as a Rare Complication of Scrub Typhus: A Case Report

**DOI:** 10.7759/cureus.42358

**Published:** 2023-07-24

**Authors:** Anadika Rana, Jay Tewari, Shubhajeet Roy, Punyagam Batra, Pratiksha Pant, Deepak Sharma, Virendra Atam, Kartikeya M Tripathi

**Affiliations:** 1 Internal Medicine, King Geroge's Medical University, Lucknow, IND; 2 Faculty of Medical Sciences, King George’s Medical University, Lucknow, IND; 3 Faculty of Medical Sciences, King George's Medical University, Lucknow, IND; 4 Internal Medicine, King George's Medical University, Lucknow, IND; 5 Internal Medicine, King George’s Medical University, Lucknow, IND

**Keywords:** orientia tsutsugamushi, tsutsugamushi, orientia, acute febrile illness, acute pancreatitis, scrub typhus

## Abstract

Scrub typhus is transmitted by the bite of chiggers (larvae) of *Leptotrombidium deliense *and is caused by the bacteria *Orientia tsutsugamushi. *Common symptoms include fever, headache, lymphadenopathy, and black eschar formation, while acute pancreatitis is a rare complication. We present the case of a 27-year-old gentleman, who presented with epigastric pain and constipation for three weeks, fever for four days, and vomiting for two days. Serum lipase, C-reactive protein, and serum amylase were significantly raised. Enzyme-linked immunosorbent assay anti-scrub typhus IgM was positive at 0.605 optical density. An abdominal computed tomography scan revealed a bulky pancreas. Mild fluid collection (50 × 60 × 65 mm) was seen in the peripancreatic region, along with moderate to gross mesenteric fat stranding. The left anterior and lateral renal conal fascia were thickened and edematous. The patient was managed with intravenous fluids, antipyretics, and intravenous doxycycline.

## Introduction

Scrub typhus is a rickettsial zoonotic disease transmitted to humans by the bite of chiggers (larvae) of trombiculid mites -* Leptotrombidium deliense*. *Orientia tsutsugamushi*, an intracellular gram-negative bacteria, is the implicated causative organism. The incubation period ranges from six to 21 days. Its spectrum of clinical manifestations can range from subclinical cases to fatalities. Typical symptoms include fever and chills, headache, body ache and myalgia, lymphadenopathy, altered mentation, rash, and black eschar formation, while acute pancreatitis is one of the rarest complications. Early diagnosis by detecting serum IgM antibodies against *O. tsutsugamushi* has proven to be beneficial [1-3]. Acute pancreatitis being a rare complication of scrub typhus infection is often not suspected, which leads to a worse prognosis in cases with this complication. Having a strong index of suspicion for acute pancreatitis in patients with scrub typhus is of paramount importance, and the rationale behind reporting this case is to highlight the same. 

## Case presentation

A 27-year-old gentleman (written informed consent has been obtained to publish his data and images in an anonymous format) presented to the Internal Medicine ward of a tertiary care university hospital in Northern India, with complaints of epigastric abdominal pain and constipation for three weeks, fever for four days, and vomiting for two days. His profession was that of a daily wage worker. General examination revealed no abnormalities apart from lymphadenopathy and tenderness in the epigastric region. Abdominal examination revealed sluggish bowel sounds. A complete blood count revealed no abnormalities. Urine examination, kidney function tests, and liver function tests were all within normal limits. Serum lipase, C-reactive protein, and serum amylase were markedly raised: 143.4 IU/L (reference interval 0-38 IU/L), 47.82 mg/L (reference interval 0-6 mg/L), and 117.6 IU/L (reference interval 31-107 IU/L), respectively. Enzyme-linked immunosorbent assay (ELISA) was reactive for hepatitis B surface antigen initially but on repeat testing it was non-reactive. IgM by Typhidot and anti-hepatitis C virus total antibodies by ELISA were negative.

Given the patient’s symptoms and higher prevalence in tropical countries, scrub typhus, malaria, *Leptospira*, and *Salmonella typhi* serologies (screening tests) were sent. All were negative except for ELISA anti-scrub typhus IgM at a test optical density of 0.605 (optical density >0.500 is taken as positive).

**Figure 1:**
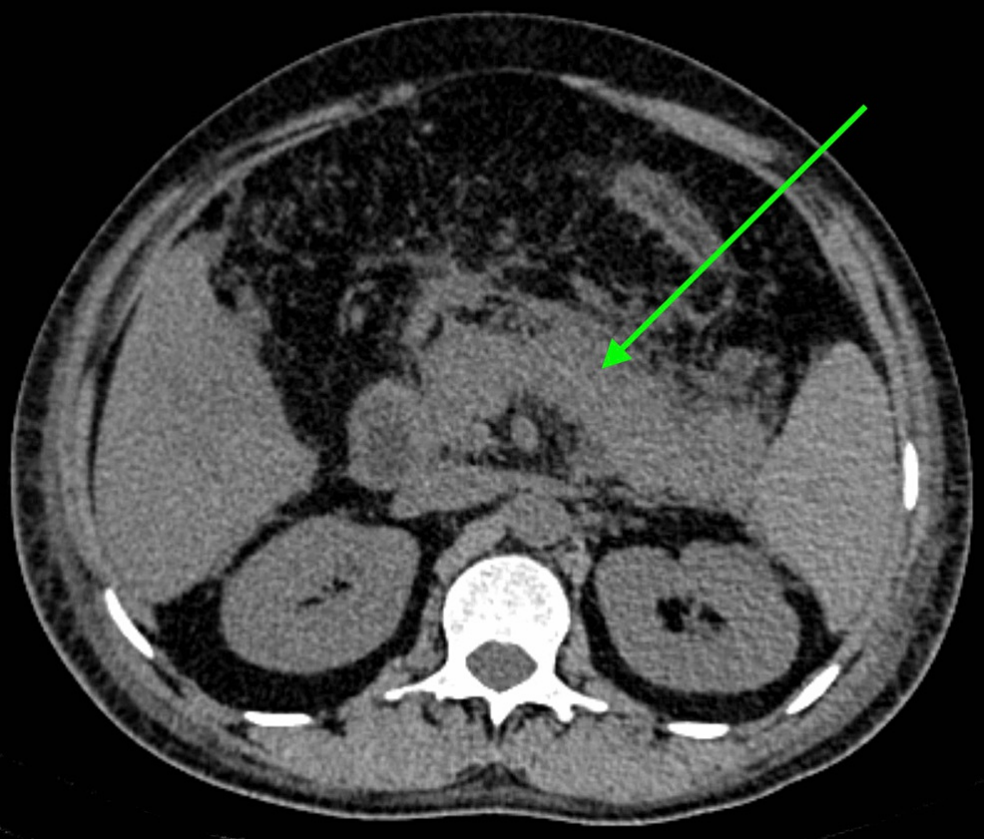
Non-contrast computed tomography of the abdomen of the patient with findings suggestive of acute pancreatitis (green arrow points toward the inflamed pancreas as seen on computed tomography)

An abdominal non-contrast computed tomography (NCCT) scan revealed a bulky pancreas, along with parenchyma of homogeneous density, and a fuzzy outline. A mild amount of fluid collection (50 × 60 × 65 mm) was seen in the peripancreatic region. Moderate to gross mesenteric fat stranding was visualized in the peripancreatic region, lesser sac, and adjacent mesentery. The left anterior and lateral renal conal fascia were thickened and edematous. The main pancreatic duct was not dilated. All the above findings were suggestive of acute pancreatitis (Figure 1).

The patient was managed conservatively with intravenous fluids along with antipyretics. He was also given intravenous doxycycline 100 mg twice daily. The patient showed improvement within two days and was started on oral feeds. The bowel movements were normal in three to four days and was discharged after eight days. A follow-up visit after 15 days showed no signs of any residual disease processes within the reference range investigations. The on-admission and follow-up significant investigations have been listed in Table 1.

**Table 1:** Comparison between on-admission and 15-day follow-up investigations. IU/L: international unit per liter; mg/dL: milligram per deciliter.

	On admission	On 15-day follow-up	Reference range
Serum lipase (in IU/L)	143.4	25.6	0-38
C-reactive protein (in mg/dL)	47.82	4.3	0-6
Serum amylase (in IU/L)	117.6	78.8	31-107

## Discussion

Scrub typhus is a mite-borne infectious disease. It is one of the main etiological agents causing acute undifferentiated febrile illness, along with leptospirosis, dengue, malaria, etc. The disease has a broad spectrum of manifestations and clinical symptoms [1]. 

There has been an upsurge in the cases of scrub typhus after years of quiescence. Ranjan J, et al. in 2018 concluded that the changes in land use, land cover, and urbanization are some of the main contributing factors behind the surge, in addition to the availability of better tests, changes in antimicrobial use, and climate change [4]. 

This recent upsurge in the number of cases makes it essential to understand the various manifestations and complications of the disease so that early diagnosis and treatment can increase the chances of complete recovery. The case series done by Ahmed et al. found a mortality rate of 42.8% in patients with acute pancreatitis complicating scrub typhus [5].

Diagnostic modalities such as IgM antibody assay by ELISA or indirect fluorescent antibody tests have been used to confirm scrub typhus conventionally. Though, nowadays newer techniques such as loop-mediated isothermal amplification assay and metagenomic next-generation sequencing are also available [6]. Diagnosis of acute pancreatitis is done based on elevated enzymes and imaging studies. Serum lipase has been reported to have a higher sensitivity and specificity of up to 82%-100%. Ultrasound can be used as an initial imaging modality owing to its lower cost, quickness, and ease of use. Computed tomography scans are better at the delineation of structural pathologies. Magnetic resonance imaging can be used in non-emergent cases for better delineation than computed tomography scans but is not favored due to the longer scan times [5].

Implementation of appropriate treatment protocols along with these rapid diagnostic assays is imperative as scrub typhus is essentially a multisystem disease and an important differential diagnosis of pyrexia of unknown origin. 

Gastrointestinal complications like nausea, vomiting, diarrhea, and hematemesis or melena, along with signs of hepatomegaly, jaundice, and abdominal pain are common [7]. Liver involvement is due to a cell-mediated immune reaction due to elevated levels of interferon-gamma and is seen frequently by deranged liver function tests [8]. Neurological compromise involving both the central and the peripheral nervous system is seen in 20% of the cases [6]. Uncommon scrub typhus-associated complications that have been reported in the literature include myocarditis, pericarditis, glomerulonephritis, acute kidney injury, acute respiratory distress syndrome, and acute acalculous cholecystitis [9].

Acute pancreatitis is commonly caused by viral agents like mumps virus, coxsackie virus, hepatitis B virus, cytomegalovirus, varicella, herpes simplex virus, and human immunodeficiency virus. Bacterial agents like *Mycoplasma*, *Leptospira*, *Legionella*, and *Salmonella* are also implicated. However, scrub typhus is rarely associated with acute pancreatitis as an infectious cause [10].

Acute pancreatitis, as a complication of scrub typhus, is rare, with the pathogenesis being largely unknown. Small vessel vasculitis and perivasculitis owing to endothelial invasion by the offending organisms are the probable hypothesis mechanisms. Although the presentation is rare, a higher mortality rate has been associated with cases of scrub typhus that are complicated with acute pancreatitis. Therefore, it is vital to be vigilant in cases when patients start presenting with acute pancreatitis-like features and always be on the lookout for early detection and treatment of acute pancreatitis in patients with scrub typhus [5].

Various antibiotics such as doxycycline, azithromycin, and chloramphenicol are used for treating scrub typhus. The use of chloramphenicol has declined due to its toxicity profile. Single-dose azithromycin (500 mg) has been shown to have similar efficacy as doxycycline (200 mg) daily for a week in mild cases [11]. In a recent multicentric randomized control trial by Varghese et al., combination therapy with intravenous doxycycline and azithromycin has been demonstrated to have a better treatment outcome than monotherapy alone with either drug in cases of severe scrub typhus [12].

## Conclusions

Acute pancreatitis, as a complication, is rare in patients with scrub typhus. Hence, a high degree of suspicion should be borne in the attending physician’s mind, while treating such patients. One should closely monitor and look out for significant clinical features, like increased levels of serum lipase, serum C-reactive protein, and serum amylase. Upon suspicion, immediate imaging in the form of an NCCT of the whole abdomen should be sent to reach a definitive diagnosis of acute pancreatitis and to start immediate dedicated treatment.
